# The $f↔\tilde{f}$ Correspondence and Its Applications in Quantum Information Geometry

**DOI:** 10.3390/e26040286

**Published:** 2024-03-26

**Authors:** Paolo Gibilisco

**Affiliations:** Department of Economics and Finance, Tor Vergata University of Rome, Via Columbia 2, 00133 Rome, Italy; paolo.gibilisco@uniroma2.it

**Keywords:** operator monotone functions, operator means, quantum Fisher information

## Abstract

Due to the classifying theorems by Petz and Kubo–Ando, we know that there are bijective correspondences between Quantum Fisher Information(s), operator means, and the class of symmetric, normalized operator monotone functions on the positive half line; this last class is usually denoted as $F_{op}$. This class of operator monotone function has a significant structure, which is worthy of study; indeed, any step in understanding $F_{op}$, besides being interesting per se, immediately translates into a property of the classes of operator means and therefore of Quantum Fisher Information(s). In recent years, the f↔f correspondence has been introduced, which associates a non-regular element of $F_{op}$ to any regular element of the same set. In terms of operator means, this amounts to associating a mean with multiplicative character to a mean that has an additive character. In this paper, we survey a number of different settings where this technique has proven useful in Quantum Information Geometry. In Sections 1–4, all the needed background is provided. In Sections 5–14, we describe the main applications of the $f↔\tilde{f}$ correspondence.

## 1. Introduction: The Chentsov Uniqueness Theorem and the Chentsov–Morozova Problem

The basic theorems of classical and quantum information geometry are categorical in character: one is the Chentsov theorem and the other one is its quantum counterpart, the Petz–Kubo–Ando (PKA) theorem.

Let us first describe the structure of the Chentsov theorem. The idea is as follows: Imagine that we want to use a family of Riemannian metrics on the family of the simplexes of probability vectors to distinguish the states (namely the probability vectors themselves). It would be natural that these metrics should contract *under the effect of noise*, namely under the effect of the morphisms of such structures, which are the stochastic maps; the distance between states could shrink if we *muddy the waters*. Let us translate this into a formal mathematical structure.

If N is a differential manifold, let us denote by $T_{\rho }N$ the tangent space to N in the point ρ∈N. In the present commutative case, we define a *Markov morphism* as a stochastic map, $T:R^{n}\to R^{k}$. Let

(1) $$\begin{array}{c} P_{n}^{1}:=\left\{\rho \in R^{n}|\sum \rho_{i}=1,\;\rho_{i}>0\right\}. \end{array}$$

The tangent space of $P_{n}^{1}$ can be naturally represented as

(2) $$\begin{array}{c} T_{\rho }P_{n}^{1}=\left\{v\in R^{n}|\sum_{i}v_{i}=0\right\}. \end{array}$$

A *monotone metric* will be any family of Riemannian metrics $g=\{g^{n}\}$ on $\left\{P_{n}^{1}\right\}$, n∈N, such that

$$g_{T(\rho )}^{m}\left(TX,TX\right)\leq g_{\rho }^{n}\left(X,X\right)$$

holds for every Markov morphism $T:R^{n}\to R^{m}$, for every $\rho \in P_{n}^{1}$ and for every $X\in T_{\rho }P_{n}^{1}$.

Let us remember that Fisher Information is the Riemannian metric on $P_{n}^{1}$, defined as

$${\langle u,v\rangle }_{\rho ,F}:=\sum_{i}\frac{u_{i}v_{i}}{\rho_{i}},\;\;u,v\in T_{\rho }P_{n}^{1}.$$

Rao was the first to realize that Fisher Information was indeed a Riemann metric on statistical models. The surprising result, as proven by Chentsov (see [1]), is as follows:

**Theorem 1.**  *There exists a unique monotone metric on $P_{n}^{1}$ (up to scalars) given by the Fisher information.*

How do we generalize this in the quantum setting? Chentsov himself and Morozova were the ones to correctly formalize this new categorical problem (see [2]).

We denote by $M_{n}$ (resp. $M_{n,sa}$), the space of complex (resp. self-adjoint) n×n matrices and define $D_{n}^{1}$ as the space of the faithful states. This means

(3) $$\begin{array}{c} D_{n}^{1}:=\left\{\rho \in M_{n,sa}|\rho >0\;,\;\mathrm{Tr}\left(\rho \right)=1\right\}. \end{array}$$

Due to the needs of quantum dynamics in the non-commutative case, a *Markov morphism* should be defined as a completely positive and trace-preserving operator $T:M_{n}\to M_{k}$. There exists a straightforward identification of $T_{\rho }D_{n}^{1}$ with the space of self-adjoint traceless matrices; namely, for any $\rho \in D_{n}^{1}$,

(4) $$\begin{array}{c} T_{\rho }D_{n}^{1}=\left\{A\in M_{n,sa}|\mathrm{Tr}\left(A\right)=0\right\}. \end{array}$$

Emphasizing the perfect analogy with the classical case, a *monotone metric* or *Quantum Fisher Information* in the non-commutative case is defined as a family of Riemannian metrics $g=\{g^{n}\}$ on $\left\{D_{n}^{1}\right\}$, n∈N, such that

$$g_{T(\rho )}^{m}\left(TX,TX\right)\leq g_{\rho }^{n}\left(X,X\right)$$

holds for every Markov morphism $T:M_{n}\to M_{m}$, for every $\rho \in D_{n}^{1}$, and for every $X\in T_{\rho }D_{n}^{1}$.

Again, we see that distances becomes shorter under *noise* effect. It is now time to see if non-commutative monotone metrics exist and how we can classify them. The Fisher metric comes from *division by ρ*, but there is no natural *division by ρ* in the quantum setting.

To solve this problem, we need more complex mathematical instruments. This is the argument contained in the following sections.

## 2. Means for Positive Numbers and the $\tilde{f}$ Function

A basic ingredient to answer the Chentsov–Morozova problem is the notion of operator means. To introduce this, we first need to understand the notion of numerical means.

**Definition 1.**  *Let $R^{+}=\left(0,+\infty \right)$. A mean for pairs of positive numbers is a function $m\left(\cdot ,\cdot \right):R^{+}\times R^{+}\to R^{+}$ such that (see [3])*

*(1) m(x,x)=x ;*

*(2) m(x,y)=m(y,x) ;*

*(3) x<y⟹x<m(x,y)<y ;*

*(4) $x<x^{\prime }\;y<y^{\prime }\;⟹\;m\left(x,y\right)<m\left(x^{\prime },y^{\prime }\right)$ ;*

*(5) m(·,·) is continuous;*

*(6) for t>0 one has m(tx,ty)=t·m(x,y).*

**Definition 2.** 
$$M_{nu}:=\left\{m\left(\cdot ,\cdot \right):R^{+}\times R^{+}\to R^{+}|m\;\mathrm{is}\;a\;\mathrm{mean}\;\right\}$$

**Definition 3.**  *$F_{nu}$ is the class of functions $f\left(\cdot \right):R^{+}\to R^{+}$ such that*

*(i) f is continuous;*

*(ii) f is monotone increasing;*

*(iii) f(1)=1;*

*(iv) $tf\left(t^{-1}\right)=f\left(t\right)$.*

**Proposition 1.**  *There is a bijection between $M_{nu}$ and $F_{nu}$ given by the formulas*

$$m_{f}\left(x,y\right):=yf\left(xy^{-1}\right)$$

$$f_{m}\left(t\right):=m\left(1,t\right)$$

**Proof.**  Straightforward.

□

Here we have some examples of means and of the associated representing function (Table 1).

**Remark 1.**  *It is possible to prove that, in the above table, the representing functions are concave and more: they are all operator concave. However, in $F_{nu}$ we also have convex functions, such as this piecewise affine function (see [4]):*

$$f\left(x\right)=\left\{\begin{array}{cc} \frac{x+3}{4}, & if\;0\leq x\leq 1,\\ \frac{3x+1}{4}, & if\;x\geq 1. \end{array}\right.$$

*Setting $f\left(0\right)=\lim_{x\to 0}f\left(x\right)$, it is straightforward to verify that each mean $m_{f}\left(\cdot ,\cdot \right)$ has a continuous extension to [0,+∞)×[0,+∞), provided by*

$$m_{f}\left(0,y\right)=f\left(0\right)\cdot y\;m_{f}\left(x,0\right)=f\left(0\right)\cdot x\;m_{f}\left(0,0\right)=0\;x,y>0.$$

*We call the functions with f(0)>0 regular and all the others non-regular. Indeed, the associated regular means have an additive character; namely, if x,y>0 then $m_{f}\left(x,0\right)>0$ and $m_{f}\left(0,y\right)>0$. On the contrary, if f(0)=0 the mean appears multiplicative, that is, $m_{f}\left(x,0\right)=0$ and $m_{f}\left(0,y\right)=0$.*

*Note that no negative connotation should be associated with the denomination non-regular*.

**Definition 4.**  *For $f\in F_{nu}$ such that f(0)>0, we set*

$$\tilde{f}\left(x\right)=\frac{1}{2}\left[\left(x+1\right)-{\left(x-1\right)}^{2}\frac{f(0)}{f(x)}\right]\;x>0.$$

It is not difficult to see that this definition associates a non-regular element, $\tilde{f}$, to a regular one, *f*.

## 3. Operator Means, Operator Monotone Functions, Quantum Fisher Information: The Petz–Kubo–Ando Theorem

We are now ready to introduce operator means. As previously, let $M_{n}:=M_{n}\left(C\right)$ be the set of all n×n complex matrices and $M_{n,sa}$ be the set of all the self-adjoint elements of $M_{n}$. We shall denote general matrices by X,Y,…, while letters A,B,… will be used for self-adjoint matrices. The Hilbert–Schmidt scalar product will be denoted by $\langle X,Y\rangle =\mathrm{Tr}\left(X^{*}Y\right)$, where the adjoint of matrix *X* is denoted by $X^{*}$. Let $D_{n}$ be the set of strictly positive elements in $M_{n,sa}$ and let $D_{n}^{1}\subset D_{n}$ be the set of strictly positive density matrices previously introduced, namely $D_{n}^{1}=\left\{\rho \in M_{n,sa}|\mathrm{Tr}\;\rho =1,\;\rho >0\right\}$. If not otherwise specified, we shall (from now on) only consider faithful states (ρ>0).

A function f:(0,+∞)→R is said to be *operator monotone (increasing)* if, for any n∈N and *A*, $B\in M_{n,sa}$ such that 0<A≤B, the inequality f(A)≤f(B) holds. A positive operator monotone function, *f*, is said to be *symmetric* if $f\left(x\right)=xf\left(x^{-1}\right)$ and *normalized* if f(1)=1.

**Definition 5.**  *$F_{op}$ is the class of functions f:(0,+∞)→(0,+∞) such that*

(*i*) *f(1)=1,*
(*ii*) *$xf\left(x^{-1}\right)=f\left(x\right)$ for x>0,*
(*iii*) *f is operator monotone.*

Note that all the functions in Section 2 (except for the counterexample in Remark 1) belong to $F_{op}\;$.

**Proposition 2.**  *All the functions in $F_{op}$ are operator concave.*

The Kubo–Ando theory of operator means [3,5,6] can be seen as the matrix version of Section 2.

**Definition 6.**  *A bivariate mean for pairs of positive operators is a function*

(A,B)→m(A,B)

*defined in and with values in positive definite operators on a Hilbert space and satisfying mutatis mutandis conditions (1) to (5) in Definition 1. In addition, the transformer inequality (see [6])*

$$Cm\left(A,B\right)C^{*}\leq m\left(CAC^{*},CBC^{*}\right)$$

*holds for positive definite A,B, and arbitrary C.*

Notice that the transformer inequality replaces (6) in Definition 1. We denote the set of matrix means as $M_{op}$ and let $Q_{op}$ be the set of the Quantum Fisher Information(s).

The fundamental result is as follows.

**Theorem 2**  (Petz, Kubo, Ando in [6,7])**.** *There are two bijections linking $F_{op}$, $M_{op}$, and $Q_{op}$, provided by the following formulas: *

f

↕

$$m_{f}\left(A,B\right)=A^{1/2}f\left(A^{-1/2}BA^{-1/2}\right)A^{1/2}.$$

↕

$${\langle A,B\rangle }_{\rho ,f}=\mathrm{Tr}\left(A\cdot m_{f}{\left(L_{\rho },R_{\rho }\right)}^{-1}\left(B\right)\right)$$

Let us rephrase the Petz–Kubo–Ando classification theorem: any operator monotone function $f\in F_{op}$ generates an operator mean $m_{f}\left(A,B\right)$, which in turn produces Quantum Fisher Information ${\langle A,B\rangle }_{\rho ,f}$ using the above formulas. There are no other operator means or QFIs; they all come from an $f\in F_{op}$, according to the above described procedure.

This explains why it is so interesting to study the structure of $F_{op}$: any understanding in this field necessarily provides us with more insight into operator means and Quantum Fisher Information(s).

This is exactly what the $f-\tilde{f}$ correspondence will produce.

## 4. The $f↔\tilde{f}$ Bijection for Operator Monotone Functions

As in Section 2, we divide the representing functions for operator means into two parts.

**Definition 7.**  *For $f\in F_{op}$, we define $f\left(0\right)=\lim_{x\to 0}f\left(x\right).$ We say that a function $f\in F_{op}$ is regular if f(0)≠0 and non-regular if f(0)=0, cf. [8,9].*

We introduce the sets of regular and non-regular functions,

$$F_{op}^{\;r}:=\left\{f\in F_{op}|f\left(0\right)\neq 0\right\},\;F_{op}^{\;n}:=\left\{f\in F_{op}|f\left(0\right)=0\right\},$$

and notice that, trivially, $F_{op}$ is the disjoint union of $F_{op}^{\;r}$ and $F_{op}^{\;n}$.

**Definition 8.**  *For $f\in F_{op}^{\;r}$, we set*

$$\tilde{f}\left(x\right)=\frac{1}{2}\left[\left(x+1\right)-{\left(x-1\right)}^{2}\frac{f(0)}{f(x)}\right]\;x>0.$$

*Set $G\left(f\right)=\tilde{f},$ cf.* [5].

**Theorem 3.**  *The correspondence $f\to \tilde{f}$ is a bijection between $F_{op}^{\;r}$ and $F_{op}^{\;n}$.*

## 5. The Inversion Formula and Wigner–Yanase–Dyson Information

**Definition 9.**  *For $g\in F_{op}^{\;n}$, we set*

(5) $$\overset{ˇ}{g}\left(x\right)=\left\{\begin{array}{cc} g^{\prime \prime }\left(1\right)\cdot \frac{{\left(x-1\right)}^{2}}{2g(x)-(x+1)}\;,\; & x\in (0,1)\cup (1,\infty ),\\ 1, & x=1. \end{array}\right.$$

*Define $H\left(g\right)=\overset{ˇ}{g}$.*

**Proposition 3.**  *If g is non-regular then $\overset{ˇ}{g}$ is regular; namely, $\overset{ˇ}{g}\in F_{op}^{\;r}$. Moreover, if $f\in F_{op}^{\;r}$ and $g\in F_{op}^{\;n}$, then*

H(G(f))=fandG(H(g))=g.

The correspondence between the WYD information (see [10]),

$$I_{\rho }^{\beta }\left(A\right)=-\frac{1}{2}\mathrm{Tr}\left(\left[\rho^{\beta },A\right]\left[\rho^{1-\beta },A\right]\right),\;0<\beta <1/2,$$

and the Quantum Fisher Information depends on the operator monotonicity of the functions

$$f_{\beta }\left(x\right)=\beta \left(1-\beta \right)\frac{{\left(x-1\right)}^{2}}{\left(x^{\beta }-1\right)\left(x^{1-\beta }-1\right)}\;0<\beta <1/2.$$

See [8,10,11] for the existing proofs. Indeed, Proposition 3 provides a new approach to the above result.

The function

$$g_{\beta }\left(x\right)=\frac{x^{\beta }+x^{1-\beta }}{2}\;0<\beta <1/2$$

is operator monotone and, moreover, $g_{\beta }\in F_{op}$ and $g_{\beta }$ is non-regular. The calculations show that ${\tilde{f}}_{\beta }=g_{\beta }$. Therefore, the function $f_{\beta }\in F_{op}^{\;r}$ for 0<β<1/2.

Here we provide the first examples of the correspondence (Table 2).

## 6. Regular QFI in Terms of Covariance

Quantum covariance is usually defined as

(6) $$\begin{array}{c} {\mathrm{Cov}}_{\rho }\left(A,B\right):=\frac{1}{2}\mathrm{Tr}\left(\rho \left(AB+BA\right)\right)-\mathrm{Tr}\left(\rho A\right)\cdot \mathrm{Tr}\left(\rho B\right), \end{array}$$

where $A=A^{*}$ and $B=B^{*}$. The above formula can be written using the arithmetic mean of the left and right multiplication operator as

(7) $$\begin{array}{c} {\mathrm{Cov}}_{\rho }\left(A,B\right):=\mathrm{Tr}\left(\left(\frac{L_{\rho }+R_{\rho }}{2}\right)\left(A_{0}\right)B_{0}\right), \end{array}$$

where $A_{0}=A-\mathrm{Tr}\left(\rho A\right)\cdot I$. This simple remark led Petz to the following definition (see [12]):

**Definition 10.**  *For any $f\in F_{\mathrm{op}}$, define the Quantum f-Covariance as*

(8) $$\begin{array}{c} {\mathrm{Cov}}_{\rho }^{f}\left(A,B\right):=\mathrm{Tr}\left(m_{f}\left(L_{\rho },R_{\rho }\right)\left(A_{0}\right)B_{0}\right). \end{array}$$

As usual, ${\mathrm{Var}}_{\rho }^{f}\left(A\right):={\mathrm{Cov}}_{\rho }^{f}\left(A,A\right)$. If f(x)=(1+x)/2, then

(9) $$\begin{array}{c} {\mathrm{Cov}}_{\rho }^{f}\left(A,B\right)=\frac{1}{2}\mathrm{Tr}\left(\rho \left(AB+BA\right)\right)-\mathrm{Tr}\left(\rho A\right)\cdot \mathrm{Tr}\left(\rho B\right)={\mathrm{Cov}}_{\rho }\left(A,B\right), \end{array}$$

which is the above given standard definition for the quantum covariance.

With this generalized notion of Petz covariance, we show that there is an unexpected relation between QFI and the covariance itself.

We stated previously that there exists a natural identification of $T_{\rho }D_{n}^{1}$ with the space of self-adjoint traceless matrices; namely, for any $\rho \in D_{n}^{1}$

$$T_{\rho }D_{n}^{1}=\left\{A\in M_{n}|A=A^{*}\;,\;\mathrm{Tr}\;A=0\right\}.$$

Moreover, the PKA theorem states that the Quantum Fisher Information(s) are given by the formula

$${\langle A,B\rangle }_{\rho ,f}=\mathrm{Tr}\left(A\cdot m_{f}{\left(L_{\rho },R_{\rho }\right)}^{-1}\left(B\right)\right)$$

for positive matrices $A,B\in T_{\rho }D_{n}^{1}$, where $f\in F_{op}$.

Monotone metrics are usually normalized in such a way that [A,ρ]=0 implies $g_{\rho }\left(A,A\right)=\mathrm{Tr}\left(\rho^{-1}A^{2}\right)$.

**Remark 2.**  Let us remember that $T_{\rho }:=\left\{A=A^{*}|\mathrm{Tr}\left(\rho A\right)=0\right\}$; the tangent space in ρ to the state space has a natural orthogonal decomposition in terms of “commuting” and “noncommuting” parts as

(10) $$\begin{array}{c} T_{\rho }=T_{\rho }^{c}⊕T_{\rho }^{n}, \end{array}$$

*where*

(11) $$\begin{array}{c} T_{\rho }^{c}=\left\{A=A^{*}|\left[\rho ,A\right]=0\right\},\;T_{\rho }^{n}=\left\{i\left[\rho ,A\right]|A=A^{*}\right\}. \end{array}$$

*Due to the Chentsov uniqueness theorem, the different QFI(s) are characterized from what they do on the noncommuting part of the tangent space; namely, on $T_{\rho }^{n}$ that is on tangent vectors of the form i[ρ,A].*

*We are now ready to state the QFI(s) in terms of covariances.*

**Theorem 4.**  *Gibilisco, Imparato, and Isola (Proposition 6.3, page 11 in [13]).*

*If $f\in F_{\mathrm{op}}^{\;r}$, then*

(12) $$\begin{array}{c} \frac{f(0)}{2}\cdot {\langle i\left[\rho ,A\right],i\left[\rho ,B\right]\rangle }_{\rho ,f}={\mathrm{Cov}}_{\rho }\left(A,B\right)-{\mathrm{Cov}}_{\rho }^{\tilde{f}}\left(A,B\right). \end{array}$$

The above formula has many important consequences.

## 7. A Look at the Petz–Sudar Theorem

In the PKA classification theorem (Theorem 2 in Section 3), we see that the QFI is defined only for faithful states (ρ>0). It is Petz himself, in collaboration with Sudár, who understood how to define a *radial extension* of a QFI to pure states and how to prove that only regular QFIs possess such an extension (for all details, the reader can refer to [9] or to [13]). The statement is as follows:

**Theorem 5**  (Petz and Sudár in [9])**.** *A QFI admits a radial extension iff it is regular (f(0)>0). In such a case*

(13) $$\begin{array}{c} 2f\left(0\right){\langle \cdot ,\cdot \rangle }_{\cdot ,f}\to {\langle \cdot ,\cdot \rangle }_{\cdot ,\mathrm{FS}} \end{array}$$

*where ${\langle \cdot ,\cdot \rangle }_{\cdot ,\mathrm{FS}}$ is the Fubini-study metric on the space of pure states.*

The fact that the radial limit of $2f\left(0\right){\langle \cdot ,\cdot \rangle }_{\cdot ,f}$ does not depend on *f* is an immediate consequence of Theorem 4 in Section 6.

It is natural to ask, can the Petz–Sudár theorem be generalized and proven using Formula (22)? Here, generalization means using Formula (22) for states that are neither faithful nor pure.

## 8. Extension of Regular QFI and MASI for Non-Faithful States

A far-reaching generalization of the Wigner–Yanase Skew Information has been proposed by Hansen in [8].

**Definition 11.**  *Metric Adjusted Skew Information (MASI).*

*For $f\in F_{\mathrm{op}}^{\;r}$ and ρ>0, set*

(14) $$\begin{array}{c} I_{\rho }^{f}\left(A\right):=\frac{f(0)}{2}\cdot {\langle i\left[\rho ,A\right],i\left[\rho ,A\right]\rangle }_{\rho ,f}. \end{array}$$

In the case where $f\left(x\right)={\left(1+\sqrt{x}\right)}^{2}/4$, we can see that the MASI coincides with the Wigner–Yanase Skew Information:

(15) $$\begin{array}{c} I_{\rho }\left(A\right):=I_{\rho }^{f}\left(A\right)=-\frac{1}{2}\mathrm{Tr}\left({\left[\sqrt{\rho },A\right]}^{2}\right). \end{array}$$

Note that recently, using MASI, it has been proven that the Local Quantum Uncertainty (LQU) and the Interferometric Power (IP), which are two important measures of quantum discord, are instances of a family of quantum discords parametrized by the function $f\in F_{\mathrm{op}}^{\;r}$. This allows a unified study of the properties of LQU and IP (see [14]). Due to Theorem 4, we have the following:

**Proposition 4.** 
(16) $$\begin{array}{c} I_{\rho }^{f}\left(A\right)={\mathrm{Var}}_{\rho }\left(A\right)-{\mathrm{Var}}_{\rho }^{\tilde{f}}\left(A\right). \end{array}$$

It is important to note that the two sides of Equation (16) are somehow different in nature. The MASI on the left side is defined only for faithful states (ρ>0), while the right-hand side always makes good sense since quantum covariance is defined for any state. Therefore, one can look to Equation (16) as a “definition” of the LHS, which solves the problem of extending the MASI with an approach that is different from the one proposed by Hansen in Theorem 3.8 in [8]. Motivated by the above consideration, it is natural to introduce the following sesquilinear form, which is the natural extension of MASI for two observables.

**Definition 12.** 
$$I_{\rho }^{f}\left(A,B\right):={\mathrm{Cov}}_{\rho }\left(A,B\right)-{\mathrm{Cov}}_{\rho }^{\tilde{f}}\left(A,B\right).$$

Another important remark is that, using the $f-\tilde{f}$ correspondence, it is possible to establish a relation between MASI and the quasi-entropy $S_{F}\left(\cdot ,\cdot \right)$ introduced by Petz in [15]; $S_{F}\left(\cdot ,\cdot \right)$ can be seen as a quantum version of Csiszar *F*-entropy in classical statistics and information theory (see [16]). Indeed, if Tr(ρA)=0, Theorem 3.1 in [17] proves that

$$\frac{\partial^{2}}{\partial t\partial s}S_{\tilde{f}}\left(\rho +ti\left[\rho ,A\right],\rho +si\left[\rho ,A\right]\right){|}_{t=s=0}=2I_{\rho }^{f}\left(A\right).$$

## 9. Inequalities for the MASI and the Bloch Sphere Case

In this section, we discuss some basic properties of MASI and we will see how the $\tilde{f}$ function appears, for example, as a calculation tool. What follows is the generalization of the work in [18] that appears in [19].

(a) If a quantum evolution is given by a Hamiltonian *H* that commutes with the observable *A*, then the MASI is a constant of motion. Namely, if we set $\rho_{H}\left(t\right):=e^{-itH}\rho e^{itH}$ and [A,H]=0, then the function $I_{\rho_{H}\left(t\right)}^{f}\left(A\right)$ is constant. Since the Quantum Fisher Information contracts under coarse graining, we can see that QFI is a unitary covariant, and this is the crucial ingredient of the proof.

(b) For any MASI, we have:

(17) $$I_{\rho }^{f}\left(A\right)\leq I_{\rho }^{SLD}\left(A\right)\leq \frac{1}{2f(0)}I_{\rho }^{f}\left(A\right).$$

(c) The constant $\frac{1}{2f(0)}$ is optimal in inequality (17). Namely, if $1\leq k<\frac{1}{2f(0)}$, the inequality

$$I_{\rho }^{SLD}\left(A\right)\leq kI_{\rho }^{f}\left(A\right)$$

is false and a counterexample can be found in the elementary 2×2 case, namely on the Bloch sphere.

Let us see how this can be proven by means of the $\tilde{f}$ function.

Let $\left\{\varphi_{i}\right\}$ be a complete orthonormal base composed of eigenvectors of ρ, and $\left\{\lambda_{i}\right\}$ the corresponding eigenvalues. Set $a_{ij}\equiv \langle A_{0}\varphi_{i}|\varphi_{j}\rangle$, where $A_{0}=A-\mathrm{Tr}\left(\rho A\right)$. Note that $a_{ij}\neq A_{ij}:=$ the i,j entry of *A*.

Recall that the Pauli matrices are as follows:

$$\sigma_{1}=\left(\begin{array}{cc} 0 & 1\\ 1 & 0 \end{array}\right),\;\sigma_{2}=\left(\begin{array}{cc} 0 & -i\\ i & 0 \end{array}\right),\;\sigma_{3}=\left(\begin{array}{cc} 1 & 0\\ 0 & -1 \end{array}\right).$$

A generic 2×2 density matrix in the Stokes parameterization is written as

$$\rho =\frac{1}{2}\left(\begin{array}{cc} 1+x & y+iz\\ y-iz & 1-x \end{array}\right)=\frac{1}{2}\left(I+x\sigma_{1}+y\sigma_{2}+z\sigma_{3}\right),$$

where $\left(x,y,z\right)\in R^{3}$ and $x^{2}+y^{2}+z^{2}\leq 1$. Let $r:=\sqrt{x^{2}+y^{2}+z^{2}}\in \left[0,1\right]$. The eigenvalues of ρ are $\lambda_{1}=\frac{1-r}{2}$ and $\lambda_{2}=\frac{1+r}{2}$.

**Proposition 5.** 
$$I_{\rho }^{f}\left(A\right)=\left[1-m_{\tilde{f}}\left(1-r,1+r\right)\right]\cdot {\left|a_{12}\right|}^{2}.$$

**Corollary 1.**  *If r≠0 then*

$$I_{\rho }^{SLD}\left(A\right)=\left[\frac{r^{2}}{1-m_{\tilde{f}}\left(1-r,1+r\right)}\right]\cdot I_{\rho }^{f}\left(A\right).$$

**Proposition 6.**  *If f is regular, then*

$$\lim_{r\to 0}\frac{r^{2}}{1-m_{\tilde{f}}\left(1-r,1+r\right)}=-\frac{1}{2{\tilde{f}}^{\prime \prime }\left(1\right)}=\frac{1}{2f(0)}.$$

From this last result, the optimality of the constant follows.

## 10. The Dynamical Uncertainty Principle

From Equation (16), one has

$${\mathrm{Var}}_{\rho }\left(A\right)\geq I_{\rho }^{f}\left(A\right).$$

This is the case n=1 of the Dynamical Uncertainty Principle, which reads

(18) $$\begin{array}{c} \det\left\{{\mathrm{Cov}}_{\rho }\left(A_{j},A_{k}\right)\right\}\geq \det\left\{I_{\rho }^{f}\left(A_{j},A_{k}\right)\right\}, \end{array}$$

or equivalency

(19) $$\begin{array}{c} \det\left\{{\mathrm{Cov}}_{\rho }\left(A_{j},A_{k}\right)\right\}\geq \det\left\{f\left(0\right)\cdot \frac{1}{2}\cdot {\langle i\left[\rho ,A_{j}\right],i\left[\rho ,A_{k}\right]\rangle }_{\rho ,f}\right\}, \end{array}$$

where $f\in F_{\mathrm{op}}^{\;r}$. On the left-hand side, we have the *Generalized Variance* of the random vector $\left(A_{1},...,A_{n}\right)$. Please note that, in this case, the right-hand side depends on the state-observables’ non-commutativity, and this is strictly related to a non-trivial dynamic induced by the observables according to the Schrödinger equation.

To understand the terminology, recall that the Standard Uncertainty Principle (SUP) in the Robertson version reads

(20) $$\begin{array}{c} \det\left\{{\mathrm{Cov}}_{\rho }\left(A_{j},A_{k}\right)\right\}\geq \det\left\{-i\cdot \frac{1}{2}\cdot \mathrm{Tr}\left(\rho \left[A_{j},A_{k}\right]\right)\right\}, \end{array}$$

where $A_{1},...,A_{n}$ is an arbitrary number of observables (self-adjoint matrices) and ρ is a state. For n=2, one obtains the Schrödinger uncertainty principle from which the Heisenberg uncertainty principle follows. The bound in the right-hand side depends on the non-commutativity among the observables (see [20,21]).

Now, let n=2m+1 be odd; in this case the right-hand side is the determinant of an antisymmetric matrix and therefore is zero; for an odd number of observables the SUP does not say anything “quantum”.

Therefore, using the QFI and the $f↔\tilde{f}$ correspondence, a new uncertainty principle has been proven, which is also not trivial for an odd number of observables. Moreover, SUP and DUP have been generalized for an arbitrary *g*-covariance; see [22,23,24].

If we set

$$V\left(f\right):=\det\{I_{\rho }^{f}\left(A_{j},A_{k}\right)\},$$

one can see that (Theorem 4.4 in [22])

$$\tilde{f}\leq \tilde{g}\;⟹\;V\left(f\right)\geq V\left(g\right).$$

This implies, for example, that we have the biggest bound in the DUP for f(x)=(1+x)/2. Indeed, in this case, $\tilde{f}\left(x\right)=2x/\left(1+x\right)\leq \tilde{g}$ for any regular *g*, and this provides the conclusion.

## 11. Semplification of Kosaki’s Work

To see how the $f-\tilde{f}$ correspondence sheds light on certain subjects, consider the paper by Kosaki [25]. In this paper, the author’s aim is to study how the RHS of the DUP (for n=2) depends on the function *f*. The main result by Kosaki is as follows. Remember that

$$f_{\beta }\left(x\right)=\beta \left(1-\beta \right)\frac{{\left(x-1\right)}^{2}}{\left(x^{\beta }-1\right)\left(x^{1-\beta }-1\right)}\;0<\beta <1/2,$$

$${\tilde{f}}_{\beta }\left(x\right)=\frac{1}{2}\left(x^{\beta }+x^{1-\beta }\right)$$

Let $\rho ,A_{1},A_{2}$ be fixed and set

$$F\left(f\right):=\det\left\{{\mathrm{Cov}}_{\rho }\left(A_{i},A_{j}\right)\right\}-\det\left\{I_{\rho }^{f}\left(A_{i},A_{j}\right)\right\}.$$

$$F\left(\beta \right):=F\left(f_{\beta }\right).$$

The main result in [25] is Theorem 5, which reads as follows: F(β) is decreasing in (0,1/2), F(1/2)≥0 so that F(β)≥0. The result was the final output of a rather complicated *tour de force* of calculations.

Look how simple the approach is using the $f↔\tilde{f}$ correspondence. First of all, it is straightforward that

$$\tilde{f}\leq \tilde{g}⟹F\left(\tilde{f}\right)\leq F\left(\tilde{g}\right).$$

For *x* fixed, the function

$$\beta \to {\tilde{f}}_{\beta }\left(x\right)=\frac{1}{2}\left(x^{\beta }+x^{1-\beta }\right)$$

is decreasing in (0,1/2) so that

$$\beta_{1}\leq \beta_{2}⟹{\tilde{f}}_{\beta_{1}}\geq {\tilde{f}}_{\beta_{2}}⟹F\left(\beta_{1}\right)\geq F\left(\beta_{2}\right),$$

and the Kosaki’s conclusion follows. One should read the complicated proof in [25] to fully appreciate the efficiency and clarity of the $f-\tilde{f}$ correspondence.

## 12. Refinements of Heisenberg Uncertainty Relations

In the literature, several quantities appear with the same aim: to measure *quantum uncertainty*. We will discuss some examples in this paper. For example, to quantify such uncertainty Luo introduced the following state-observable quantity,

$$U_{\rho }\left(A\right):=\sqrt{V_{\rho }{\left(A\right)}^{2}-{\left(V_{\rho }\left(A\right)-I_{\rho }\left(A\right)\right)}^{2}},$$

where $V_{\rho }\left(A\right):={\mathrm{Var}}_{\rho }\left(A\right)$. Furthermore, he was able to prove the following inequality:

$$U_{\rho }\left(A\right)\cdot U_{\rho }\left(B\right)\geq \frac{1}{4}{\left|\mathrm{Tr}\left(\rho [A,B]\right)\right|}^{2}.$$

Clearly, this can be seen as a refinement of the Heisenberg uncertainty principle because ${\mathrm{Var}}_{\rho }\left(A\right)\geq U_{\rho }\left(A\right)$.

After some failed attempt to generalize this result (see Kosaki [25], Remarks 3.2 and 3.3), Yanagi (see [26]) was able to prove a generalization that makes sense for the WYD information. He introduced the following quantity,

$$U_{\rho }^{\beta }\left(A\right):=\sqrt{V_{\rho }{\left(A\right)}^{2}-{\left(V_{\rho }\left(A\right)-I_{\rho }^{\beta }\left(A\right)\right)}^{2}},$$

and was able to prove this inequality:

$$U_{\rho }^{\beta }\left(A\right)\cdot U_{\rho }^{\beta }\left(B\right)\geq \beta \left(1-\beta \right){\left|\mathrm{Tr}\left(\rho [A,B]\right)\right|}^{2}.$$

Note that $\beta \left(1-\beta \right)=f_{\beta }\left(0\right)$ where

$$f_{\beta }\left(x\right)=\beta \left(1-\beta \right)\frac{{\left(x-1\right)}^{2}}{\left(x^{\beta }-1\right)\left(x^{1-\beta }-1\right)}\;0<\beta <1/2,$$

which is the function associated with the WYD information. It is straightforward to propose an *f*-depending quantity,

$$U_{\rho }^{f}\left(A\right):=\sqrt{V_{\rho }{\left(A\right)}^{2}-{\left(V_{\rho }\left(A\right)-I_{\rho }^{f}\left(A\right)\right)}^{2}},$$

as a measure of quantum uncertainty and try to prove the following inequality:

$$U_{\rho }^{f}\left(A\right)\cdot U_{\rho }^{f}\left(B\right)\geq f\left(0\right){\left|\mathrm{Tr}\left(\rho [A,B]\right)\right|}^{2}\;f\in F_{\mathrm{op}}^{\;r}.$$

Unfortunately, this inequality, in general, is false. Yanagi proved that the theorem holds true under a condition involving $\tilde{f}$; namely, we have the following:

**Proposition 7.**  *For $f\in F_{\mathrm{op}}^{\;r}$ if*

$$\frac{x+1}{2}+\tilde{f}\left(x\right)\geq 2f\left(x\right)\;x>0$$

*then it holds*

$$U_{\rho }^{f}\left(A\right)\cdot U_{\rho }^{f}\left(A\right)\geq f\left(0\right)\cdot \mathrm{Tr}\left(\rho \left[A,B\right]\right)\;f\in F_{\mathrm{op}}^{\;r}$$

On the other hand, one can prove the following:

**Proposition 8.**  *For any $f\in F_{\mathrm{op}}^{\;r}$ and x>0*

$$\tilde{f}{\left(x\right)}^{2}\leq \frac{1}{4}{\left(x+1\right)}^{2}-f{\left(0\right)}^{2}{\left(x-1\right)}^{2}.$$

This has the following as a consequence:

**Corollary 2.** 
$$f{\left(0\right)}^{2}{\left(x-y\right)}^{2}\leq \frac{1}{4}{\left(x+y\right)}^{2}-m_{\tilde{f}}{\left(x,y\right)}^{2}$$

From this, an unconditional inequality follows: if we switch from the constant f(0) to the constant $f{\left(0\right)}^{2}$ (see [27]), we obtain the following:

**Proposition 9.**  *For $f\in F_{\mathrm{op}}^{\;r}$ and $A,B\in M_{n,sa}$, it holds that*

$$U_{\rho }^{f}\left(A\right)\cdot U_{\rho }^{f}\left(A\right)\geq f{\left(0\right)}^{2}\cdot \mathrm{Tr}\left(\rho \left[A,B\right]\right).$$

## 13. State Quantum Uncertainty Based on MASI

Luo proposed a notion of quantum uncertainty depending only on the state ρ. In the paper [28], starting from the Wigner–Yanase information and from an orthonormal basis $\left\{H_{j}\right\}$, he introduced the quantity

$$Q^{WY}\left(\rho \right):=\sum_{j}I_{\rho }^{WY}\left(H_{j}\right)$$

as a measure of such uncertainty. First, Luo proved that $Q^{WY}\left(\rho \right)$ is basis independent, and after that

$$Q^{WY}\left(\rho \right)=\sum_{j<k}{\left(\sqrt{\lambda_{j}}-\sqrt{\lambda_{k}}\right)}^{2},$$

where $\left\{\lambda_{j}\right\}$ is the spectrum of ρ. Applications of the function $Q^{WY}\left(\rho \right)$ also appear in paper [29].

If we remember that the WY information is the QFI associated with the functions

$$f_{WY}\left(x\right):={\left(\frac{1+\sqrt{x}}{2}\right)}^{2},\;\;{\tilde{f}}_{WY}=\sqrt{x},$$

we obtain

$$Q^{WY}\left(\rho \right)=2\sum_{j<k}\left[\frac{\lambda_{j}+\lambda_{k}}{2}-\sqrt{\lambda_{j}\lambda_{k}}\right]=2\sum_{j<k}\left[m_{a}\left(\lambda_{j},\lambda_{k}\right)-m_{{\tilde{f}}_{WY}}\left(\lambda_{j},\lambda_{k}\right)\right].$$

The above considerations lead naturally to the following questions:

(i) For a regular $f\in F_{op}^{r}$, does the definition

$$Q^{f}\left(\rho \right):=\sum_{j}I_{\rho }^{f}\left(H_{j}\right)$$

produce a basis-independent function of the state ρ?

(ii) Imagine that we obtain a positive answer for (i). We may also ask if

$$Q^{f}\left(\rho \right)=2\sum_{j<k}\left[m_{a}\left(\lambda_{j},\lambda_{k}\right)-m_{\tilde{f}}\left(\lambda_{j},\lambda_{k}\right)\right].$$

These questions both received a positive answer from Cai in their paper [30]. Once again, the $\tilde{f}$ function shows up when one has to look at a general scheme for Quantum Fisher Information.

## 14. The Average Coherence of a Quantum State

In a recent paper [31], Fan, Li, and Luo attempted to study quantum coherence (an important feature of a quantum system) by eliminating the influence of a reference basis. They introduced the average quantum coherence using three procedures: (1) average over all orthonormal basis; (2) average over all elements of operator orthonormal basis; (3) average over a complete family of MUBs (Mutually Unbiased Bases). The result of the paper was that these three different procedures produce the same quantity. The basic ingredient of the proof is the $f-\tilde{f}$ correspondence.

Indeed, if E is a quantum channel and $\left\{E_{j}\right\}$ are the Kraus operators of E, the authors define a channel-depending coherence as

$$C_{f}\left(\rho ,E\right):=\sum_{j}I_{\rho }^{f}\left(E_{j}\right).$$

In the first case, they consider the channel as induced by a von Neumann measurement or equivalently by an orthonormal basis. Averaging on this reference basis is equivalent to integrating over the unitary orbit of a fixed basis, which amounts to using the normalized Haar measure over the unitary group, U, of the system Hilbert space. They set

$$C_{f}^{U}\left(\rho \right)=\int_{U}C_{f}\left(\rho |U\Pi U^{†}\right)dU,$$

where $U\Pi U^{†}=\{U|i\rangle \langle i|U^{†}:i=1,2,...,d\}$. In the second case, they defined

$$C_{f}^{\mathrm{ob}}\left(\rho \right)=\frac{1}{d+1}\sum_{\alpha =1}^{d^{2}}I_{\rho }^{f}\left(X_{\alpha }\right),$$

where $\left\{X_{\alpha }:\alpha =1,2,...,d^{2}\right\}$ is a family of $d^{2}$ operators that constitute an operator orthonormal basis for L(H), the space of all bounded linear operators on *H*. This can be proven to be independent of the chosen basis.

In the third case, they define the $C_{f}^{\mathrm{mub}}\left(\rho \right)$ coherence averaging on MUBs, which surely exist if *d* is a power of a prime number.

Finally, they proved the following result:

**Theorem 6.**  *For any state ρ of any prime power dimensional system and for any regular operator monotone function f, one has that*

$$C_{f}^{U}\left(\rho \right)=C_{f}^{\mathrm{ob}}\left(\rho \right)=C_{f}^{\mathrm{mub}}\left(\rho \right)=\frac{d-\mathrm{Tr}[m_{\tilde{f}}\left(L_{\rho },R_{\rho }\right)]}{d+1}.$$

Note that if $\lambda_{i}$ are the eigenvalues of the state ρ, then $\mathrm{Tr}\left[m_{\tilde{f}}\left(L_{\rho },R_{\rho }\right)\right]=\sum_{ij}m_{\tilde{f}}\left(\lambda_{i},\lambda_{j}\right).$

## 15. Conclusions

The notion of means has its roots deeply situated in the history of Western mathematics; the Greeks themselves knew eleven different types of means. Still, the subject is currently undergoing strong developments. As an example, starting from the work of Rao [32,33] and Prakasa–Rao [34,35], the Jensen inequality for numerical and operator means has been proven, and more generalizations seem to be on their way [4,36,37].

The $f↔\tilde{f}$ correspondence is indeed a correspondence between means. From the work by Petz, we know that two of the basic objects of Quantum Probability and Quantum Statistics, namely Quantum Covariance and Quantum Fisher Information, are indeed necessarily built on the notion of operator mean, and this explains why we find the manifold of different applications of the $f↔\tilde{f}$ correspondence described in this paper.

It is rational to expect that this is not the end of the story and that many other applications will appear in the coming years.

At the moment, the most promising area is that of the extension of the Petz–Kubo–Ando theorem for states that are neither faithful nor pure. We expect this could be performed using the formula

(21) $$\begin{array}{c} \frac{f(0)}{2}\cdot {\langle i\left[\rho ,A\right],i\left[\rho ,B\right]\rangle }_{\rho ,f}={\mathrm{Cov}}_{\rho }\left(A,B\right)-{\mathrm{Cov}}_{\rho }^{\tilde{f}}\left(A,B\right) \end{array}$$

for regular *f*. The right-hand side makes sense for any state and, on the other hand, by a continuity-approximation argument the formula is, somehow, forced to be unique; indeed, we can approximate any state by faithful states. A fully satisfying theorem would certainly deduce the scalar product

(22) $$\begin{array}{c} {\mathrm{Cov}}_{\rho }\left(A,B\right)-{\mathrm{Cov}}_{\rho }^{\tilde{f}}\left(A,B\right) \end{array}$$

from first principles, as in the Petz proof of the PKA theorem. At the moment, a similar theorem has not yet been proven. 

## Figures and Tables

**Table 1:** Means and representing function.

Name of the mean	*f*	$m_{f}$
Arithmetic	$\frac{1+x}{2}$	$\frac{x+y}{2}$
Heinz	$\frac{1}{2}\left(x^{\beta }+x^{1-\beta }\right)$	$\frac{1}{2}\left(x^{\beta }y^{1-\beta }+x^{1-\beta }y^{\beta }\right)$
	β∈(0,1/2)	β∈(0,1/2)
Geometric	$\sqrt{x}$	$\sqrt{xy}$
Logarithmic	$\frac{x-1}{\log x}$	$\frac{x-y}{\log x-\log y}$
Harmonic	$\frac{2x}{x+1}$	$\frac{2}{\frac{1}{x}+\frac{1}{y}}$

**Table 2:** $f↔\tilde{f}$ Correspondence.

*f*	$\tilde{f}$
$\frac{1+x}{2}$	$\frac{2x}{x+1}$
${\left(\frac{1+\sqrt{x}}{2}\right)}^{2}$	$\sqrt{x}$
$\beta \left(1-\beta \right)\frac{{\left(x-1\right)}^{2}}{\left(x^{\beta }-1\right)\left(x^{1-\beta }-1\right)}$	$\frac{x^{\beta }+x^{1-\beta }}{2}$

where β∈(0,1/2).
